# Dietary patterns of Chinese puerperal women and their association with postpartum weight retention: Results from the mother–infant cohort study

**DOI:** 10.1111/mcn.13061

**Published:** 2020-07-21

**Authors:** Niuniu Li, Xiao Su, Tan Liu, Jing Sun, Yimin Zhu, Zhiyong Dai, Yanchun Zhang, Lina Pan, Wei Jiang, Wenli Zhu

**Affiliations:** ^1^ Department of Nutrition and Food Hygiene Peking University School of Public Health Beijing China; ^2^ National Health Commission Key Laboratory of Reproductive Health Peking University Beijing China; ^3^ Peking‐Ausnutria Maternal and Infant Nutrition Research Center Peking University Beijing China

**Keywords:** dietary pattern, postpartum weight retention (PPWR), principal component analysis (PCA), puerperal women

## Abstract

Dietary intake may affect maternal health, but it remains unclear about puerperal dietary intake and its association with maternal health. This study investigated the dietary patterns and their related factors and association with postpartum weight retention (PPWR) in Chinese puerperal women. Participants were from the mother–infant cohort study, in which both mothers and infants were followed up from childbirth to the next 2 years, in seven cities around China. Maternal puerperal dietary patterns were derived by a food frequency questionnaire and principal component analysis (PCA) within 1 month postpartum. PPWR was assessed by the difference of weight at 42 days and 6 months postpartum minus the pre‐pregnancy weight. Of 503 postpartum women, four dietary patterns were identified, including ‘plant food’ pattern (rice and vegetables as dominant foods), ‘diverse’ pattern (starchy roots, fruit, livestock meat and aquatic products), ‘traditional northern’ pattern (poultry, eggs and soup) and ‘marine‐flour’ pattern (flour, coarse food grains and marine fish). The diverse pattern was associated with professional puerperal family care and counselling service (*p* < .05). PPWRs at 42 days and 6 months postpartum were 6.37 and 4.70 kg averagely. The plant food dietary pattern tended to be associated with higher 42‐day PPWR (β = .105, *p* < .05), and diverse pattern was associated with lower 6‐months PPWR (β = −.137, *p* < .05). Conclusively, this study presented four dominant dietary patterns in Chinese postpartum women and showed a lower PPWR in adherence to diverse dietary pattern. The results would provide evidence to furtherly guide dietary practice and improve maternal health.

**Key Messages**

- Four dietary patterns within 1 month postpartum were identified in Chinese puerperal women by principal component analysis, including ‘plant food’ pattern (rice and vegetables), ‘diverse’ pattern (starchy roots, fruit, livestock meat and aquatic products), ‘traditional northern’ pattern (poultry meat, eggs and soup) and ‘marine‐flour’ pattern (flour, coarse food grains and marine fish).
- Postpartum dietary patterns were associated with maternal sociodemographic characteristics and puerperal care and service. The diverse dietary pattern was positively associated with professional puerperal care and counselling service.
- Dietary patterns were associated with postpartum weight retention. The plant food pattern was associated with higher 42‐day PPWR, and the diverse pattern was associated with lower 6‐month PPWR.

## 1 INTRODUCTION

Countries vary in the beliefs, practices and customs related to childbearing and motherhood manners. In China, within 30–40 days after birth, women participate in a group of traditional practices referred to ‘zuoyuezi’ or ‘doing the month’ in Chinese, including eating special foods, staying in warmth and restricting physical and outdoor activities (Ding et al., 2019). The practices were supposed to emanate from a long‐held respect for tradition, elders and cultural beliefs. The consumption of specific foods is believed to play a major role during the postpartum period (Holroyd, Lopez, & Chan, 2011). According to the dual principle of *yin* and *yang* in traditional Chinese medicine, puerperal women are considered to exhaust yang. They are prescribed yang foods considered to be warm in inherent nature (not temperature) to restore balance and strength, compensate for blood loss, increase breast milk production and decrease blood clots and ‘cold wind (yin)’ in the body, including eggs, animal viscera, pork feet, millet, chicken or fish soup, noodles, various herbs and brown sugar. Inversely fresh fruit, vegetables, cold foods and even seafood, which are considered to be ‘cold’, are always restricted (Holroyd et al., 2011; Liu, Petrini, & Maloni, 2015). However, the traditional practices are characterized by high intake of poultry, egg and soups and low intake of fresh fruit and vegetables that do not fit current evidence‐based dietary guidelines well (Bao et al., 2010). Traditional practices seem to be believed nearly by all elder Chinese population, but recently, some new mothers have not deferred to the advice of their elders to completely follow the traditional diet (Ding, Yu, Vinturache, Gu, & Lu, 2018; Zheng, Watts, & Morrell, 2019). Dietary patterns in Chinese postpartum women remain unclear and complicated, mixed by traditional value and modern nutrition guidelines. Many other factors have an influence on postpartum dietary practices, such as residence region, family income, maternal educational level, caregivers, medical professionals service and health education attendance (Bao et al., 2010).

Besides offspring development, pregnancy has been reported as a crucial point of intervention for maternal health during the life course (Hill, Skouteris, & Fuller‐Tyszkiewicz, 2013; van der Pligt et al., 2013). Childbearing contributes to obesity in women, with many of them who gain excessive weight remaining overweight permanently (Davis & Olson, 2009). In high‐income countries, the proportion of women with substantial postpartum weight retention (≥4.55 kg) ranges from 14% to 25% (Lipsky, Strawderman, & Olson, 2012; Olson, Strawderman, Hinton, & Pearson, 2003). According to the Chinese National Nutrition and Health Surveillance (2010–2013), the prevalence of severe postpartum weight retention (PPWR, ≥5 kg) was 41.5% (Wang et al., 2016). Excessive PPWR is also a potential risk factor for further noncommunicable diseases such as diabetes, hypertension and cardiovascular diseases and even affected subsequent pregnancy outcomes (Nehring, Schmoll, Beyerlein, Hauner, & von Kries, 2011; Rooney, Schauberger, & Mathiason, 2005; Tahir et al., 2019; Villamor & Cnattingius, 2006). The causal contributors to PPWR are biopsychosocially complex, such as gestational weight gain, diet and exercise (Dalrymple, Flynn, Relph, O'Keeffe, & Poston, 2018; He et al., 2015; Rong et al., 2015). Postpartum dietary intake may be associated with weight retention and gain. A systematic review and meta‐analysis has evaluated the effectiveness of interventions against PPWR and indicated that nutritional intervention was significantly more effective in PPWR (−3.25 kg averagely, ranged from −4.69 to −1.82 kg), compared with routine nurse care or other interventions (Vincze et al., 2019);

Dietary intake of postpartum women may be associated with maternal health and breast milk composition, and even affect the health and development of children (Bravi et al., 2016; Koletzko et al., 2019; Lopez‐Olmedo et al., 2016; Tuokkola et al., 2016). Using dietary patterns to assess dietary quality and its association with diseases has become increasingly common, which may avoid the well‐documented limitations of the single‐food or nutrient approach (Previdelli, de Andrade, Fisberg, & Marchioni, 2016). In general, two approaches to assess dietary patterns are used: a priori, based on prior nutrition knowledge translated into dietary guidelines and a posteriori, where patterns are defined once the dietary intake data are collected. Principal component analysis (PCA) is an a posteriori approach, which more accurately captures overall dietary exposure and allows evaluation of the cumulative and interactive effects between dietary factors (Hearty & Gibney, 2013; Smith, Emmett, Newby, & Northstone, 2013). Dietary patterns, especially the optimal dietary pattern, remain unclear in Chinese postpartum women.

Therefore, this study assessed the maternal dietary patterns by PCA and investigated their related factors and association with PPWR in Chinese postpartum women. The results would provide scientific evidence to furtherly guide mothers' postpartum dietary practice and improve maternal health.

## 2 MATERIALS AND METHODS

### 2.1 Participants

The data used in this study were collected from part of the mother–infant cohort study (MICS) of China. The MICS was conducted from June 2015 to December 2018 in seven cities (Beijing, Taiyuan of Shanxi, Jinan of Shandong, Changsha of Hunan, Shiyan of Hubei and Chongqing and Chengdu of Sichuan) from northern, eastern, central and south‐western districts around China. At baseline, the inclusion criteria of participants were as follows: (a) giving birth to one child within 3 months; (b) aged ≥18 years when pregnant; (c) without serious illness; and (d) living in those cities for more than 5 years. Women whose child was born with severe deformities or dysplasia were excluded. The study was approved by the Peking University Institutional Review Board (Beijing, China), and written informed consent documents were obtained from all mother participants.

At baseline, 540 mother–infant pairs at 0–3 months postpartum were voluntarily recruited from local child care clinics and then followed up at 6–8, 12–14, 18–20 and 24–26 months postpartum, respectively. The data of 0–3 and 6–8 months postpartum were analysed, including diet and weight information. Questionnaires with >10% of the Food Frequency Questionnaire (FFQ) data missing were considered invalid, and 503 participants were included in this study at the end.

### 2.2 Data collection

#### 2.2.1 Dietary intake

Maternal dietary intake was assessed using an existing validated FFQ adapted from the Chinese National Nutrition and Health Surveillance (2010–2013). The original FFQ had 17 food groups, including nine groups of fluids intake (different soups and drinks), that were thought important for maintaining lactation in Chinese traditional dietary culture (Liu et al., 2015). This study focused on the maternal health outcome (PPWR), so the original food groups were not fully suitable. On the other hand, the ‘Chinese Dietary Guideline’ was revised in 2016, in which some foods were recommended specially for puerperal women. Based on the above consideration, the original 17 food groups were modified to 15 groups in this study, namely, rice, flour, coarse food grains, starchy roots, dark vegetables, light vegetables, fruit, livestock meat, poultry meat, eggs, aquatic products, marine fish, milk, legumes products and soup, according to their nutritional significance, revised dietary guideline and puerperal women's eating habits. The original nine groups of different soups and drinks were summarized into one ‘soup’ group. The original one staple food group was divided into four groups of rice, flour, whole grain and starchy roots, respectively. The original one vegetable group was divided into two groups, dark and light vegetables, because dark vegetables are rich in carotenoid (pro‐vitamin A), which is recommended at a much higher intake in puerperal women according to the Chinese Dietary Guideline 2016. Marine fish rich in n‐3 polyunsaturated fatty acid (DHA), which plays an important role in breastfed infant's brain development (Bourre & Paquotte, 2008), was divided from other aquatic animal products. The ‘marine fish’ referred to the seawater fish only, and the ‘aquatic products’ were identified as the aquatic animal products, including fish, shrimp, shellfish and crabs, except seawater fish.

Participants were asked to recall the quantity and frequency of food they consumed within 1 month postpartum (not the past 1 month) at the baseline (0–3 months postpartum) or the second (6–8 months) follow‐up survey. The food portion serial images, drawn on the coordinate axis, were referred to help the participants to estimate the food portion size. The quantity (g) was reported according to frequencies of consumption (never, day, week and month), and daily food intakes were calculated by multiplying the frequency of consumption for portion size (g). Then dietary patterns were extracted by PCA using the weight (g) of each food group consumed.

#### 2.2.2 Postpartum weight retention

Maternal prepregnant and postpartum weight (kg) were collected by questionnaire. The weight of pre‐pregnancy and delivery were acquired from the data record from the antenatal examination. Women's weight at 42 days postpartum were recalled by participants at the baseline or the second follow‐up survey. The weight at 6 months postpartum were measured by the investigator of the study. PPWRs were calculated as the weight at 42 days and 6 months postpartum minus the pre‐pregnancy weight. And gestational weight gain equalled to the weight before delivery minus the pre‐pregnancy weight.

#### 2.2.3 Related factors

The related factors were investigated using self‐reported questionnaire at baseline, including age, monthly family income per capita (yuan), maternal education level, delivery mode (vaginal or caesarean delivery), main family caregivers of women during puerperium (mother herself, husband, puerpera's parents or parents‐in‐law and maternity matron), whether to receive professional dietary counselling, pre‐pregnancy body mass index (BMI) and infant feeding pattern (breast feeding or mixed feeding). ‘Exclusive breastfeeding’ was defined that the infant within 6 months was fed exclusively by breast milk without anything else, except the medicine and nutrients supplementation. ‘Mixed feeding’ was defined that the infant within 6 months was fed by breast milk and infant formula. Pre‐pregnant BMI was estimated according to the weight and height measured at the first antenatal care during pregnancy 12–13 weeks. The investigation was conducted by trained investigators (local doctors or nurses) according to a uniform procedure manual. The finished questionnaire was checked, and the missing items were verified.

### 2.3 Statistical analysis

Principal component analysis was used to examine dietary patterns. Principal factors were linear combinations of the input variables and explained as much of the variation in the data as possible. Each factor described a dietary pattern, and the higher the pattern scored, the more likely it was present in the respondents' diet. All 15 food groups were input in the analysis. Sample suitability was assessed by using the Kaiser–Mayer–Olkin test and Bartlett's test of sphericity. According to the Kaiser criterion, remaining dietary patterns were determined based on the screen plot, eigenvalues (>1) and interpretability of each factor, which were calculated by a standardized matrix. Varimax rotation was conducted to highlight the interpretation of each factor. The dietary pattern described by each factor was interpreted by factor loadings, which were the correlation coefficients between factors and each input food group. Factor loadings with an absolute value ≥.4 were considered to characterize dietary patterns. Each factor scores were calculated by summing the products of multiplying the consumption and their factor loadings of each significant food group. And the higher scores of each factor (dietary patterns) means more adherence to the dietary pattern. The factor scores of each dietary pattern were normally continuous variables; however, the subgroups of the dietary pattern were more meaningful than the scores themselves, and the tertiles of factor score distribution were generally analysed according to the participants' characteristics. According to factor scores distribution, adherence to each dietary pattern was categorized as T1 (first tertile), T2 (second tertile) and T3 (third tertile).

Variable description was used to characterize the population using frequencies or means. Chi‐squared test was adapted to compare the categorical variables. Multiple linear regression was conducted to analyse the association between PPWR and related factors. The independent variables were selected according to the results of univariate analysis of the study and other related studies (He et al., 2015; Rong et al., 2015; Widen et al., 2015), and the following variables were included: dietary pattern (factor scores, as continuous variables), pre‐pregnancy BMI, gestational weight gain (GWG), infant feeding pattern, maternal age, educational level, family monthly income per capita (yuan), delivery mode, caregiver during puerperium and professional dietary counselling. Two‐sided *p* < .05 denoted statistical significance.

### 2.4 Ethical considerations

This study was conducted according to the guidelines laid down in the Declaration of Helsinki, and all procedures involving research study participants were approved by the Peking University Institutional Review Board (Beijing, China). Written informed consent was obtained from all subjects.

## 3 RESULTS

### 3.1 Characteristics of participant women

Overall, most of the participants (85.4%) were aged 21–34 years. Among all 503 participants, 60.6% were highly educated (bachelor's degree or higher), and 63.0% were mainly cared for by their parents or parents‐in‐law during puerperium. The characteristics of the participants are shown in Table 1.

**TABLE 1:** Characteristics of the 504 postpartum women^†^

Characteristic	*n*	%
Age group, yo		
21–29	218	43.3
30–34	212	42.1
35–45	73	14.5
Educational level		
Bachelor's degree or higher	305	60.6
Junior college	99	19.7
High school or lower	99	19.7
Monthly family income per capita, yuan		
≤3,000	99	21.7
3,000–6,000	187	41.0
≥6,000	170	37.3
Delivery mode		
Vaginal	287	58.0
Caesarean	208	42.0
Puerperal caregiver of women		
Puerpera herself	54	11.1
Husband	38	7.8
Puerpera's parents	307	63.0
Maternity matron	88	18.1
Professional dietary counselling		
Yes	235	46.7
No	268	53.3
Infant feeding pattern		
Breast feeding	245	48.7
Mixed feeding	258	51.3
Postpartum period, month(s)		
1–	154	30.6
2–	291	57.9
3–	58	11.5
Total	503	100.0

^†^ Total number for each category does not always equal the number of participants due to missing data, including delivery mode, monthly family income per capita and main family caregivers of women.

### 3.2 Identification of dietary patterns within 1 month postpartum

**FIGURE 1:**
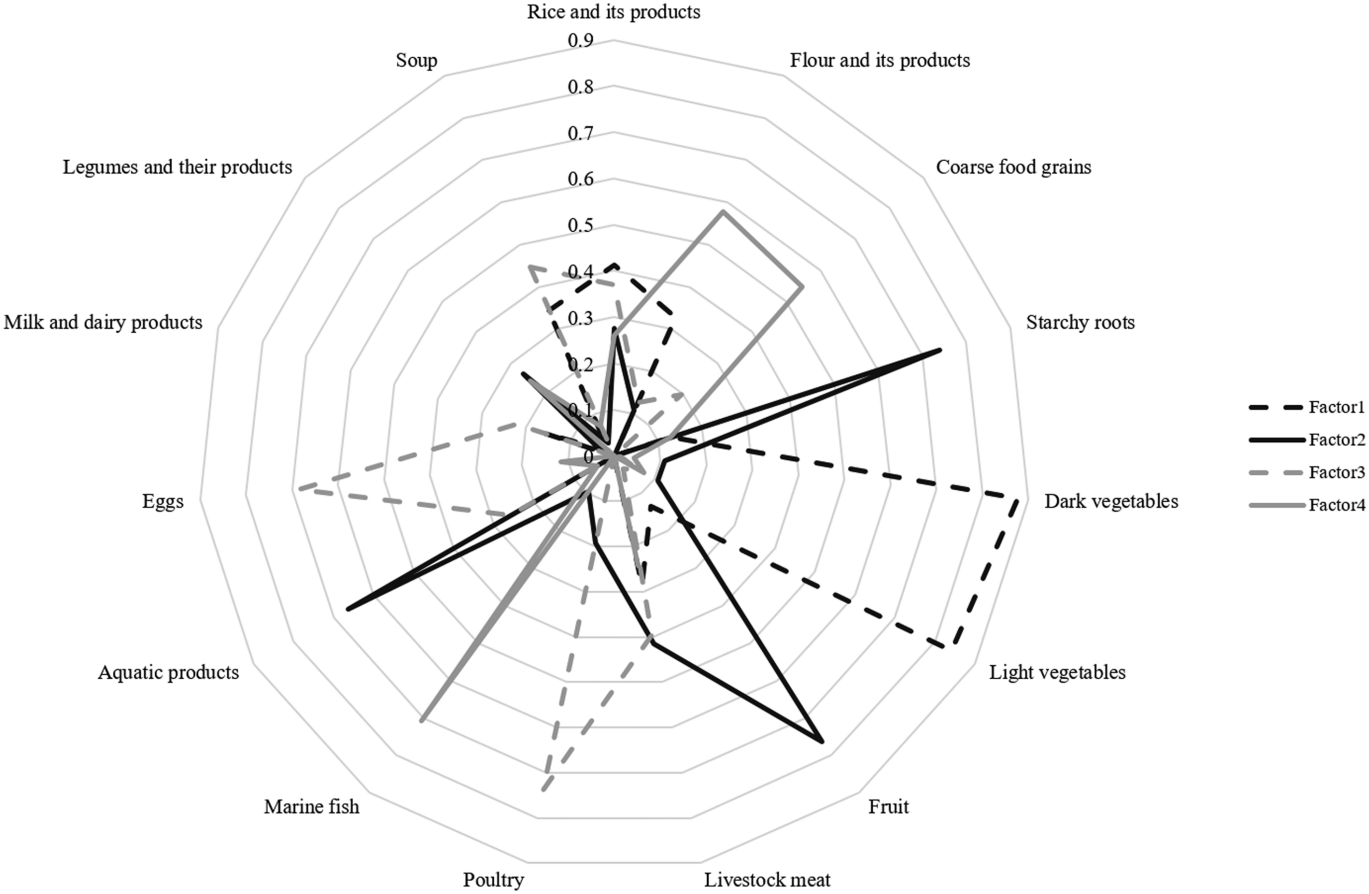
Factor loadings that characterize each dietary pattern

Four dietary patterns were identified in postpartum women by the PCA (Table 2). The eigenvalues were 1.929, 1.922, 1.598 and 1.416, and the patterns explained 12.056%, 12.011%, 9.989% and 8.849% of variance among food groups, respectively. Factor 1 (‘plant food’ pattern) was characterized by high loadings for rice and its products, dark vegetables and light vegetables. Factor 2 (‘diverse’ pattern) was characterized by high loadings for starchy roots, fruit, livestock meat and aquatic products other than sea fishes. Factor 3 (‘traditional northern’ pattern) was characterized as high intake of poultry, eggs and soup, which was a typical Chinese tradition dietary practice during doing the month (puerperal) period. Factor 4 (‘marine‐flour’ pattern) was characterized by high loadings for flour and its products, coarse food grains and marine fish. The details are shown in Table 2 and Figure 1.

**TABLE 2:** Factor loadings for food groups in varimax‐rotated principal components^†^

	Factor 1	Factor 2	Factor 3	Factor 4
Eigenvalue	1.929	1.922	1.598	1.416
% variance explained	12.056	12.011	9.989	8.849
Food groups				
Rice and its products	0.413	0.276	0.371	0.261
Dark vegetables	0.876	0.109	0.016	0.042
Light vegetables	0.841	0.108	0.059	0.073
Starchy roots	0.129	0.739	−0.062	0.129
Fruit	0.134	0.762	0.034	−0.098
Livestock meat	0.287	0.416	0.395	0.243
Aquatic products	−0.026	0.664	0.258	0.043
Poultry	0.002	0.193	0.739	−0.036
Eggs	−0.022	−0.069	0.685	0.116
Soup	0.345	0.030	0.447	0.071
Flour and its products	0.327	0.107	0.127	0.579
Coarse food grains	−0.042	−0.070	0.197	0.547
Marine products	0.035	0.094	−0.166	0.708
Milk and dairy products	0.160	0.026	0.222	−0.152
Legumes and their products	0.017	0.265	−0.067	0.244

^†^ Factor 1 (‘plant food’ pattern), Factor 2 (‘diverse’ pattern), Factor 3 (‘traditional northern’ pattern) and Factor 4 (‘marine‐flour’ pattern).

### 3.3 Related factors of postpartum dietary patterns

As shown in Table 3, the dietary patterns of participants were significantly related with sociodemographic characteristics, puerperal caregiver and professional counselling service. The dietary pattern of participants cared for by a maternity matron during puerperium was more likely to be in the highest tertile of Factor 1 (plant food pattern), compared with those being cared for by other caregivers (χ^2^ = 15.882, *p* < .05), and a lower score of Factor 1 was presented in elderly parturient women (≥35 years; χ^2^ = 10.002, *p* < .05). The higher adherence to Factor 2 (diverse pattern) was associated with being care for by a maternity matron (χ^2^ = 13.548, *p* < .05) and counselling service (χ^2^ = 6.402, *p* < .05). A higher score of Factor 3 (traditional northern pattern) was associated with younger age (<30 years; χ^2^ = 13.768, *p* < .05), and puerperal care (husbands and parents as caregiver; χ^2^ = 17.167, *p* < .05). A higher score of Factor 4 (marine‐flour pattern) was associated with lower family income (monthly family income per capita <3,000 yuan; χ^2^ = 21.167, *p* < .05).

**TABLE 3:** Characteristics of postpartum women based on the tertiles of factor scores of dietary patterns^†^ *n* (%)

	Factor 1 (plant food pattern)			Factor 2 (diverse pattern)			Factor 3 (traditional northern pattern)			Factor 4 (marine‐flour pattern)		
	T1	T2	T3	T1	T2	T3	T1	T2	T3	T1	T2	T3
Age group, yo												
21–29	85(39.0)	59(27.1)	74(33.9)	68(31.2)	73(33.5)	77(35.3)	64(29.4)	66(30.3)	88(40.4)	74(33.9)	62(28.4)	82(37.6)
30–34	58(27.4)	80(37.7)	74(34.9)	74(34.9)	73(33.4)	65(30.7)	69(32.5)	80(37.7)	63(29.7)	68(32.1)	79(37.3)	65(30.7)
35–45	24(32.9)	29(39.7)	20(27.4)	25(34.2)	23(31.5)	25(34.2)	34(46.6)	23(31.5)	16(21.9)	25(34.2)	28(38.4)	20(27.4)
χ^2^		10.022			1.346			13.768			5.639	
*p*		.040^*^			.854			.008^*^			.228	
Educational level												
Bachelor's degree or higher	100(32.8)	102(33.4)	103(33.8)	98(32.1)	97(31.8)	110(36.1)	103(33.8)	102(33.4)	100(32.8)	108(35.4)	106(34.8)	91(29.8)
Junior college	34(34.3)	29(29.3)	36(36.4)	36(36.4)	35(35.4)	28(28.3)	28(28.3)	37(37.4)	34(34.3)	33(33.3)	27(27.3)	39(39.4)
High school or lower	33(33.3)	37(37.4)	29(29.3)	33(33.3)	37(37.4)	29(29.3)	36(36.4)	30(30.3)	33(33.3)	26(26.3)	36(36.4)	37(37.4)
χ^2^		1.796			3.138			1.847			6.113	
*p*		.773			.535			.764			.191	
Monthly family income per capita (yuan)												
≤3,000	34(34.3)	33(33.3)	32(32.3)	44(44.4)	27(27.3)	28(28.3)	32(32.3)	32(32.3)	35(35.4)	25(25.3)	26(26.3)	48(48.5)
3,000–6,000	60(32.1)	62(33.2)	65(34.8)	63(33.7)	65(34.8)	59(31.6)	68(36.4)	53(28.3)	66(35.5)	58(31.0)	65(34.8)	64(34.2)
≥6,000	52(30.6)	58(34.1)	60(35.3)	45(26.5)	63(37.1)	62(36.5)	53(31.2)	70(41.2)	47(27.6)	72(42.4)	60(35.3)	38(22.4)
χ^2^		0.473			9.300			7.121			21.167	
*p*		.976			.054			.130			<.001^*^	
Delivery mode												
Vaginal	92(32.1)	95(33.1)	100(34.8)	91(31.7)	95(33.1)	101(35.2)	93(32.4)	102(35.5)	92(32.1)	94(32.8)	89(31.0)	104(36.2)
Caesarean	68(32.7)	73(35.1)	67(32.2)	73(35.1)	71(34.1)	64(30.8)	70(33.7)	65(31.3)	73(35.1)	72(34.6)	78(37.5)	58(27.9)
χ^2^		0.404			1.164			1.050			4.201	
*p*		.817			.559			.592			.122	
Puerperal caregiver of women												
Puerpera herself	24(44.4)	20(37.0)	10(18.5)	16(29.6)	20(37.0)	18(33.3)	14(25.9)	26(48.1)	14(25.9)	16(29.6)	24(44.4)	14(25.9)
Husband	10(26.3)	16(42.1)	12(31.6)	16(42.1)	14(36.8)	8(21.1)	16(42.1)	8(21.1)	14(36.8)	12(31.6)	12(31.6)	14(36.8)
Puerpera's parents	111(36.2)	96(31.3)	100(32.6)	111(36.2)	100(32.6)	96(31.3)	93(30.3)	102(33.2)	112(36.5)	111(36.2)	103(33.6)	93(30.3)
Maternity matron	19(21.6)	29(33.0)	40(45.5)	17(19.3)	31(35.2)	40(45.5)	41(46.6)	26(29.5)	21(23.9)	26(29.5)	24(27.3)	38(43.2)
χ^2^		15.882			13.548			17.167			8.763	
*p*		.014^*^			.035^*^			.009^*^			.187	
Professional dietary counselling												
Yes	75(31.9)	81(34.5)	79(33.6)	66(28.1)	80(34.0)	89(37.9)	76(32.3)	86(36.6)	73(31.1)	75(31.9)	82(34.9)	78(33.2)
No	92(34.3)	87(32.5)	89(33.2)	101(37.7)	89(33.2)	78(29.1)	91(34.0)	83(31.0)	94(35.1)	92(34.3)	87(32.5)	89(33.2)
χ^2^		0.377			6.402			1.884			0.440	
*p*		.828			.041^*^			.390			.803	

^*^ *p <* .05.

^†^ T1 was the lowest tertile of factor score, T2 was the medium tertile of factor score and T3 was the highest of factor score.

### 3.4 Relations of dietary patterns with PPWR

PPWRs at 42 days and 6 months postpartum were on average 6.37 and 4.70 kg, respectively. Mean PPWRs according to different dietary patterns are shown in Figures **S2** and **S3**.

Multivariable linear regression analysis showed that, as shown in Table 4, the higher 42‐day PPWR was associated with higher plant food pattern score (β = .105, *p* < .05). The 6‐month PPWR was lower in women adhering to diverse dietary pattern (β = −.137, *p* < .05). Both the 42‐day and 6‐month PPWRs were higher in women with higher GWG (β = .706, *p* < .05; β = .208, *p* < .05) and increased as pre‐pregnancy BMI decreased (β = −.110, *p* < .05; β = −.322, *p* < .05).

**TABLE 4:** Results of the multiple linear regression analysis between PPWR and related factors

Independent variables	42‐day PPWR			6‐month PPWR		
	β	95% CI	*p*	β	95% CI	*p*
Factor 1 (plant food pattern) score	.105	(0.154, 0.796)	.004^*^	.088	(−0.136, 1.142)	.123
Factor 2 (diverse pattern) score	−.049	(−0.562, 0.103)	.176	−.137	(−2.211, −0.210)	.018^*^
Factor 3 (traditional northern pattern) score	−.004	(−0.340, 0.307)	.920	−.109	(−1.376, 0.012)	.054
Factor 4 (marine‐flour pattern) score	.068	(−0.016, 0.663)	.062	−.053	(−0.970, 0.361)	.369
Pre‐pregnancy BMI	−.110	(−0.327, −0.065)	.004^*^	−.322	(−1.092, −0.512)	<.001^*^
Gestational weight gain (GWG)	.706	(0.628, 0.772)	<.001^*^	.208	(0.121, 0.414)	<.001^*^
Infant feeding pattern	−.046	(−1.175, 0.248)	.201	.111	(−0.026, 3.019)	.054
Maternal age	.058	(−0.105, 0.946)	.116	.033	(−0.818, 1.471)	.575
Educational level	.028	(−0.293, 0.654)	.455	.115	(−0.016, 1.998)	.054
Monthly family income per capita (yuan)	−.038	(−0.745, 0.239)	.313	.011	(−0.958, 1.156)	.854
Delivery mode	−.016	(−0.875, 0.554)	.658	019	(−1.291, 1.826)	.736
Puerperal caregiver	−.038	(0.154, 0.796)	.294	−.015	(−0.992, 0.761)	.795
Professional dietary counselling	−.016	(−0.562, 0.103)	.675	−.011	(−1.680, 1.379)	.847

*Note.* BMI, body mass index; CI, confidence interval; PPWR, postpartum weight retention.

* *p* < .05.

## 4 DISCUSSION

Of Chinese postpartum women, four dietary patterns were identified through a posteriori PCA, including plant food pattern (characterized dominantly by rice and vegetables), diverse pattern (starchy roots, fruit, livestock meat and aquatic products), traditional northern pattern (poultry meat, eggs and soup) and marine‐flour pattern (flour, coarse food grains and marine fish). According to the Chinese Dietary Guidelines (2016), postpartum women are recommended to follow a diverse diet including a broad spectrum of nutritious foods, which is similar to the diverse pattern derived in the study. But the food groups of other derived dietary patterns were less than diverse pattern. Actually, the adherence to doing the month (puerperal) practices was moderately high among Chinese women (Liu et al., 2015), especially those unhealthy dietary habits, such as eating excess animal foods and soups and restricting intake of fruits and vegetables, which is similar to the traditional northern pattern of the study.

Maternal dietary quality is crucial for both maternal and child health, but few studies focus on it. According to the dietary guidelines, there are both similarities and differences between dietary patterns of puerperal women and the general population. Puerperal women during lactation are recommended to consume a little more animal foods (fish, livestock and poultry meat, egg and dairy products) compared with the general population. Previous studies investigated the dietary patterns of Chinese general adults using data from the China Health and Nutrition Survey and identified two dietary patterns: the ‘traditional’ pattern (rice, vegetables and meat) and ‘modern’ pattern (fast food, fried products and cakes; Batis et al., 2016; Li & Shi, 2017; Shi et al., 2018). Obviously the dominant food groups in the traditional pattern were similar to that in the plant food pattern derived in the study, and the modern pattern involved a high intake of fast food and fried products, which was not identified in our study. This might be attributed to our focus on the nutrients in the food, rather than food processing or cooking methods. Another study identified three major dietary patterns in general adults: the ‘western’ pattern (beef, lamb, dairy products, soft beverages and cakes), ‘northern’ pattern (wheat flour products and starchy tubers) and ‘southern’ pattern (fruit, pork, poultry, rice, vegetables and aquatic products; Wang et al., 2011). Among these, the northern pattern corresponded to the marine‐flour pattern of the study, except for the low consumption of fishes. A possible reason was that lactating women were recommended to consume more marine products rich in polyunsaturated fatty acids, which were essential for infants' brain development. In addition to these dietary patterns presented in both general and puerperal population, the study identified a typical puerperal dietary pattern and the traditional northern pattern, characterized by animal foods (poultry meat and eggs) and soup dominantly, which followed the Chinese traditional doing the month (puerperal) practices, but was seldom in the general population. Overall, the postpartum dietary patterns identified in the study were not totally similar to those in the general population.

Maternal dietary intake would meet the requirements of both mothers and fetus or breastfed infants, in which higher intakes of animal foods (livestock, poultry meat, eggs and milk) were especially recommended and always overeaten. A study of 305 lactating women in South Central China identified two dietary patterns: Pattern 1 characterized by red meat, coarse cereals and fresh vegetables (leafy) and Pattern 2 characterized by fresh vegetables (nonleafy), soy milk and bacteria and algae. The mean energy, protein, fat and carbohydrate intakes of the highest quartile of both patterns exceeded the recommended nutrient intake (RNI), whereas the intakes of calcium, selenium and vitamins A, B_1_ and C were much lower than RNI (Huang, Li, & Hu, 2019). In this study, the nutrients intake has not been analysed among different dietary patterns, and the principal food components included were mainly high caloric, especially in traditional northern pattern. In order to assess the dietary quality fully and analyse their association with health outcome, the nutrients intake should be investigated furtherly, and a priori approach needs to be performed through healthy diet index assessment.

Many factors could influence postpartum dietary practices, such as culture, beliefs, sociodemographic, residence, lifestyle, puerperal caregivers, professional service, health education and pregnancy‐related determinants (Doyle, Borrmann, Grosser, Razum, & Spallek, 2017). Our study found that postpartum dietary patterns were significantly associated with maternal age, educational level, family income, puerperal family care and organizational counselling service. More professional care (maternity matron as caregiver) and counselling service were positively related with the diverse pattern (relatively healthy diet), inversely with the traditional northern pattern (*p* < .05). And the marine‐flour pattern was more likely to exist in low‐income families (*p* < .05). Among these related factors, the organizational counselling service is the adjustable and available factor, especially for guiding postpartum women towards healthier rather than unhealthier dietary pattern behaviours. Many studies showed that culturally sensitive postpartum care and health education about the practices could facilitate the immediate and long‐term health of the mothers and infants. Liu et al. (2009) put forward a content outline for postpartum education that could help Chinese women alter their food choices, in a culturally sensitive and non‐judgemental manner, through providing not only the common knowledge but also the cultural understanding. The educators might also need to include family members into health education because they have a major influence on the postpartum women. A randomized controlled trial showed that postpartum nutritional education could significantly improve the Chinese women's nutrition and dietary intake (Liu et al., 2009).

Postpartum weight retention could increase the risks for maternal noncommunicable diseases and subsequent pregnancy (Haugen et al., 2014; Widen et al., 2015). This study furtherly analysed the association between dietary patterns and PPWR and then showed that being more adhering to the plant food pattern was related to higher 42‐day PPWR, whereas the diverse pattern could decrease 6‐month PPWR. The results demonstrated that the diverse pattern was a relatively healthy dietary pattern and could improve maternal health. Higher carbohydrate intake as suggested by the ‘plant pattern’ (i.e., rice) may lead to higher weight retention, but this still needs to be investigated further. Meta‐analysis and systematic review showed that combined interventions of diet and physical activities could reduce PPWR in women (Farpour‐Lambert, Ells, Martinez De Tejada, & Scott, 2018), and nutritional intervention was significantly more effective on improving postpartum weight outcomes compared with usual care or other interventions (Vincze et al., 2019).

This study also showed that lower pre‐pregnancy BMI and higher GWG were associated with higher PPWR. However, not all women with higher pre‐pregnancy BMI have lower GWG, one possible explanation was it did not mean that higher pre‐pregnancy BMI was beneficial to postpartum weight reduction, and women with lower BMI were more likely to gain more weight during pregnancy because of weight gain recommendations than women with higher BMIs. Subsequently, women with lower BMI may have a harder time reducing their PPWR. Therefore, GWG may mediate the relationship between pre‐pregnancy BMI and PPWR.

Even so, our study could not draw conclusion that higher pre‐pregnancy BMI was beneficial to postpartum weight reduction. The possible reason is that women with higher pre‐pregnancy BMI always have lower GWG, and systematic review and meta‐analysis drew consistent conclusions and indicated that excessive GWG, rather than pre‐pregnancy BMI, determined short‐ and long‐term PPWR (Rong et al., 2015). So the results recommended that the women should have appropriate GWG according to pre‐pregnant weight, in order to reduce postpartum weight.

## 4.1 Limitations

There were some limitations in the study. Firstly, large regional differences exist in the rate of Chinese puerperal practices. Despite the participants were from different districts around China, the sampling method was not randomized, and the cohort sample size was small; thus, the generalization of the conclusion is limited. Secondly, some data (weight at 42 days postpartum and food intake within 1 month postpartum) were recalled by participants; thus, the recall bias could influence the results. Thirdly, in the study, an a posteriori approach was used to assess the dietary. Based on this, a priori dietary quality assessment approach can be used in future research, to develop a Chinese postpartum healthy eating index.

## 5 CONCLUSIONS

Four dietary patterns within 1 month postpartum were identified in Chinese puerperal women by PCA, and the plant food dietary pattern tended to be associated with higher PPWR, and the diverse pattern was associated with lower PPWR. The results provided scientific evidence to furtherly guide the postpartum dietary practice and suggested that puerperal women should adhere to a healthy diet consisting of diverse foods, which was beneficial to maternal health. However, postpartum diet could have short‐ and long‐term effects on maternal and children's health and development (Bravi et al., 2016; Dror & Allen, 2018), which should be explored furtherly and broadly, not only postpartum weight retention.

## CONFLICTS OF INTEREST

The authors declare that they have no conflicts of interest.

## CONTRIBUTIONS

NL, XS, TL, JS and WZ performed the research. WZ, ZD, YZ, LP and WJ designed the research study. WZ, YZ and WJ contributed essential reagents or tools. NL, JS and TL analysed the data. NL and XS wrote the paper.

## Supporting information


**Figure S2.** Mean PPWRs at 42 days postpartum in tertiles of dietary patterns' scores (kg) ^†^
^†^Factor 1 (‘plant food’ pattern), Factor 2 (‘diverse’ pattern), Factor 3 (‘traditional northern’ pattern), Factor 4 (‘marine‐flour’ pattern).

**Figure S3.** Mean PPWRs at 6 months postpartum in tertiles of dietary patterns' scores (kg) ^†^
^†^Factor 1 (‘plant food’ pattern), Factor 2 (‘diverse’ pattern), Factor 3 (‘traditional northern’ pattern), Factor 4 (‘marine‐flour’ pattern).
